# Predicting COVID 19–Associated Pulmonary Aspergillosis Risk in Low- and Middle-Income Countries: A Matched Case-Control Study

**DOI:** 10.1093/ofid/ofae406

**Published:** 2024-07-23

**Authors:** Merlin Moni, Dipu T Sathyapalan, Fabia Edathadathil, M Abdul Razak, Sivapriya G Nair, Chithira V Nair, Swathy S Samban, Preetha Prasanna, Kiran G Kulirankal, Shyam Sundar Purushothaman, Georg Gutjahr, Jiang Ying, Teny M John

**Affiliations:** Division of Infectious Diseases, Department of General Medicine, Amrita Institute of Medical Science and Research Centre, Amrita Vishwa Vidyapeetham, Kochi, Kerala, India; Division of Infectious Diseases, Department of General Medicine, Amrita Institute of Medical Science and Research Centre, Amrita Vishwa Vidyapeetham, Kochi, Kerala, India; Department of Infection Control and Epidemiology, Amrita Institute of Medical Science and Research Centre, Amrita Vishwa Vidyapeetham, Kochi, Kerala, India; Division of Infectious Diseases, Department of General Medicine, Amrita Institute of Medical Science and Research Centre, Amrita Vishwa Vidyapeetham, Kochi, Kerala, India; Division of Infectious Diseases, Department of General Medicine, Amrita Institute of Medical Science and Research Centre, Amrita Vishwa Vidyapeetham, Kochi, Kerala, India; Division of Infectious Diseases, Department of General Medicine, Amrita Institute of Medical Science and Research Centre, Amrita Vishwa Vidyapeetham, Kochi, Kerala, India; Division of Infectious Diseases, Department of General Medicine, Amrita Institute of Medical Science and Research Centre, Amrita Vishwa Vidyapeetham, Kochi, Kerala, India; Department of Medical Administration, Amrita Institute of Medical Science and Research Centre, Amrita Vishwa Vidyapeetham, Kochi, Kerala, India; Division of Infectious Diseases, Department of General Medicine, Amrita Institute of Medical Science and Research Centre, Amrita Vishwa Vidyapeetham, Kochi, Kerala, India; Department of Anaesthesiology and Critical Care, Amrita Institute of Medical Science and Research Centre, Amrita Vishwa Vidyapeetham, Kochi, Kerala, India; Center for Research in Analytics and Technology for Education, Amrita Vishwa Vidyapeetham, Kollam, Kerala, India; Division of Internal Medicine, Department of Infectious Diseases, Infection Control and Employee Health, The University of Texas MD Anderson Cancer Center, Houston, Texas, USA; Division of Internal Medicine, Department of Infectious Diseases, Infection Control and Employee Health, The University of Texas MD Anderson Cancer Center, Houston, Texas, USA

**Keywords:** aspergillosis, COVID-19, galactomannan, invasive fungal infection, secondary bacterial infections

## Abstract

**Background:**

Coronavirus disease 2019 (COVID-19)–associated pulmonary aspergillosis (CAPA) is a life-threatening fungal infection. Studies focusing on CAPA in low- and middle-income countries are limited.

**Methods:**

This retrospective matched case-control study was conducted at a tertiary care center in South India. Cases of CAPA were classified using the 2020 European Confederation of Medical Mycology/International Society for Human and Animal Mycology consensus criteria. A total of 95 cases were matched 1:1 with COVID-19 patients without CAPA. Matching was done based on age and period of admission. Inverse probability weighting was used to account for imbalances in COVID-19 severity and intensive care unit (ICU) admission. Data on demographics, clinical details, microbiologic and radiologic data, and treatment outcomes were collected. A predictive score for CAPA was developed from baseline risk factors.

**Results:**

The predictive score identified lymphopenia, European Organisation for Research and Treatment of Cancer risk factors, and broad-spectrum antibiotic use as the main risk factors for CAPA. Positivity for bacterial pathogens in blood or bronchoalveolar lavage samples reduced the risk of CAPA. The predictive model performed well in cross-validation, with an area under the curve value of 82%. CAPA diagnosis significantly increased mortality and shift to ICU.

**Conclusions:**

The predictive model derived from the current study offers a valuable tool for clinicians, especially in high-endemic low- and middle-income countries, for the early identification and treatment of CAPA. With further validation, this risk score could improve patient outcomes.

## Teaser

In low- and middle-income country settings, a model that incorporates risk factors such as lymphopenia, any European Organisation for Research and Treatment of Cancer risk factor, the use of broad-spectrum antimicrobials and absence of positive bacterial cultures from blood or respiratory secretions is a useful tool for predicting COVID-19–associated pulmonary aspergillosis.

The emergence of coronavirus disease 2019 (COVID-19), caused by the severe acute respiratory syndrome coronavirus 2 (SARS-CoV-2), has catalyzed a global health crisis, profoundly affecting >775 million people and resulting in >7 million deaths [1]. A significant complication in the clinical trajectory of hospitalized COVID-19 patients, especially those developing severe acute respiratory distress syndrome and requiring intensive care unit (ICU) admission, is the occurrence of secondary infections [2, 3]. Notably, COVID-19–associated pulmonary aspergillosis (CAPA), a life- threatening fungal infection caused by *Aspergillus* species, has been increasingly recognized as a major complication in critically ill COVID-19 patients. This coinfection has high morbidity rates and causes strain on the existing healthcare system [4, 5].

The diagnostic approach to CAPA has evolved substantially since its recognition as a distinct clinical entity. Initially reliant on criteria for invasive aspergillosis, the diagnosis of CAPA was rapidly tailored to address the unique interplay between SARS-CoV-2 infection and aspergillosis [6, 7]. This led to the adaptation of existing aspergillosis scores and criteria, reflecting the distinctive pathophysiology and clinical presentation of CAPA in the context of COVID-19 [8, 9]. Concurrently, the development of CAPA-specific classification criteria, such as the European Confederation of Medical Mycology/International Society for Human and Animal Mycology (ECMM/ISHAM) consensus, provided a structured framework for diagnosing CAPA, considering factors unique to COVID-19 such as immune dysregulation and severe respiratory distress [10]. The pandemic further complicated CAPA diagnosis by blurring the distinction between secondary bacterial pneumonia and invasive pulmonary mold infection, which have overlapping clinical and radiologic features at presentation. This complexity highlighted the necessity for CAPA-specific diagnostic criteria and scoring systems, designed to navigate the intricacies of CAPA and facilitate timely and accurate clinical decision-making [11, 12]. The classification system for CAPA that is most widely used in clinical scenarios today is based on the 2020 ECMM/ISHAM consensus, which classifies CAPA into possible, probable, and proven based on clinical, radiologic, and microbiologic criteria [10]. This classification requires microbiologic or histologic confirmation of invasiveness from sterile samples for proven cases, which is very challenging in COVID-19 patients owing to the risk of transmission from the aerosol-generating procedures involved [10].

Incidence of CAPA varies by geographic location. India has a higher baseline rate of invasive fungal infections (invasive candidiasis and COVID-19–associated mucormycosis); however, studies exploring CAPA in India are limited [13]. Low- and middle-income countries (LMICs) such as India face additional challenges in the form of diagnostic limitations [14] and resource constraints [15]. Oftentimes, diagnosis is delayed in these countries owing to smaller numbers of lower respiratory samples collected via bronchoalveolar lavage (BAL), scarcity of resources, and higher turnaround time for diagnostic biomarkers such as galactomannan assay. This delay could lead to detrimental outcomes for patients, emphasizing the urgent need for prompt evaluation and robust diagnostic strategies that could help in early and appropriate administration of therapeutics [9].

The objective of the current study, conducted at a tertiary care center in South India, was to investigate the risk factors, and outcomes of CAPA in an LMIC. Through a retrospective matched case-control study design, we aimed to identify predictive factors for CAPA diagnosis and to develop a clinical scoring system for stratifying patients according to the risk of CAPA. Bedside risk scoring systems could guide the prompt administration of antifungal therapy and may contribute to better outcomes.

## METHODS

### Study Setting

Our retrospective single-center case-control study analyzed patients admitted to a 1300-bedded tertiary care academic hospital in Kerala, South India, between June 2020 and June 2021. During this time, local prevalent strains of COVID-19 included Delta, Alpha, Kappa, and other strains [16].

Ethics approval was duly obtained from the Institutional Ethics Committee (IEC-AIMS-2021-GENMED-293). The study does not include factors necessitating patient consent.

### Sample Selection

All patients admitted during the period to the COVID-19 ward or ICU with active infection or those diagnosed with COVID-19 in the preceding weeks and exhibiting worsening respiratory symptoms with suspected invasive fungal infection were screened for inclusion in our analysis [10]. Among these, we included patients with suspected invasive pulmonary aspergillosis. To identify patients with suspected invasive pulmonary aspergillosis, we conducted retrospective data mining of electronic health records of COVID-19 patients for service orders for mold-active antifungals, fungal diagnostics including potassium hydroxide staining and cultures of respiratory samples, polymerase chain reaction (PCR) of respiratory samples, biomarker test (galactomannan) and chest computed tomography (CT) scans. Patients identified through any of the above service orders were further screened using the 2020 ECMM/ISHAM consensus criteria. As per institutional protocol, patients with clinical deterioration of suspected infective origin underwent sequential diagnostic sampling of blood and respiratory secretions for cultures prior to antimicrobial administration. BAL sampling was the preferred mode of diagnostic sampling of respiratory secretions in mechanically ventilated patients. In nonventilated patients, expectorated sputum or expressed sputum was sent for microscopy and cultures along with blood cultures. Patients with a history of invasive aspergillosis prior to their COVID-19 diagnosis or *Aspergillus* colonization without invasion were excluded from the study. “Invasive” disease was implied based on the clinician's discretion for treatment with mold-active antifungals, relying on radiological findings, biomarker results, and histopathological evidence of tissue invasion. Patients who had positive bacterial cultures from either blood, respiratory secretions (BAL/non-BAL), or both were classified as having a culture-positive bacterial infection in blood/respiratory secretions.

### CAPA Case Definitions

Suspected CAPA was defined as COVID-19–positive patients who had deterioration in respiratory parameters and underwent evaluation by radiology (chest X-ray/high-resolution CT), galactomannan assay, sampling of respiratory secretions (BAL/sputum) for microbiological cultures/microscopy for fungal elements/PCR, or commencement of mold-active antifungal therapy.

Cases of CAPA were classified as “proven,” “probable,” or “possible,” as defined by the 2020 ECMM/ISHAM consensus criteria [10].

“Proven” CAPA is defined as pulmonary or tracheobronchial infection proven by histopathological or direct microscopic detection, or both, of fungal elements consistent with *Aspergillus* species, showing invasive growth into tissues with associated tissue damage, or (with or without) *Aspergillus* recovered by culture or detected by microscopy/histology studies or PCR from material that was obtained by a sterile aspiration or biopsy from a pulmonary site. “Probable CAPA” was defined as having 1 of the following conditions: presence of new cavitary lung lesion(s) on chest CT without alternative explanation, positive serum galactomannan ≥1, or positive culture for *Aspergillus* species in BAL.

“Possible CAPA” included at least 1 of the following conditions: radiological signs suggestive of invasive fungal disease or culture with growth of *Aspergillus* species in non-BAL respiratory samples, which included sputum and endotracheal aspirates. The time of CAPA diagnosis was defined as the earliest date on which the diagnostic feature was identified.

For every case, an age-matched control was selected in a 1:1 ratio. Controls were COVID-19 patients without a diagnosis of invasive aspergillosis. The admission period was also matched between cases and controls to account for the circulating variants of SARS-CoV-2.

### Data Collection

Data were collected through retrospective review of the electronic medical records. The baseline data collected included demographics, risk factors, clinical details, microbiologic data, radiologic data, and treatment details. The outcome data collected included discharge status, progression to mechanical ventilation, 28-day mortality, length of ICU stay, and length of hospitalization.

### Statistical Analysis

Categorical variables were summarized by contingency tables, and quantitative variables were summarized by means and standard deviations. The χ^2^ test was used to assess differences in categorical variables, and Mann-Whitney *U* test was used to assess differences in quantitative variables. Association between binary variables between lymphopenia and immunosuppression and between broad-spectrum antibiotics and CAPA were assessed using McNemar test. The patient outcomes mechanical ventilation, transfer to ICU, and death were analyzed using Cox regression models with a time-varying covariable CAPA diagnosis. A risk score for the prediction of CAPA was developed from all the baseline characteristics using least absolute shrinkage and selection operator (LASSO). To account for the imbalance of COVID-19 severity and location of admission, a propensity score was developed using a logistic regression model and used for inverse probability weighting in LASSO. The shrinkage parameter λ for LASSO was selected by cross-validation. Exact postselection confidence intervals for LASSO were calculated after variable selection [17].

The risk score was generated from the log odds ratios in the model by multiplying to whole integers. Discriminative ability of the clinical predictive scoring was assessed by area under the receiver operating characteristic curve. The predictive cut-off value of the risk score for the presence of CAPA was assessed using the Youden index J statistic to determine an optimal threshold for CAPA risk prediction [18]. Performance measures of the selected model were calculated by 100 times, 10-fold cross-validation. Diagnostic accuracy parameters of sensitivity, specificity, and the Brier score were estimated. CAPA risk categories, comprising high and low risk, were assigned to patients in the validation cohort based on the clinical prognostic scoring system developed, and diagnostic accuracy parameters were calculated. Subgroup analysis was performed with patients who were initially admitted to ICU. The analysis was repeated with and without possible CAPA patients. In the multivariable model for the calculation of the propensity score, the dependent variable is COVID-19 severity while for the risk score the dependent variable is COVID-19–associated pulmonary aspergillosis. All statistical analyses for the study were performed in R version 4.3.1 (R Foundation for Statistical Computing, Vienna, Austria), and *P* < .05 was considered statistically significant for all analyses.

## RESULTS

**Figure 1.**
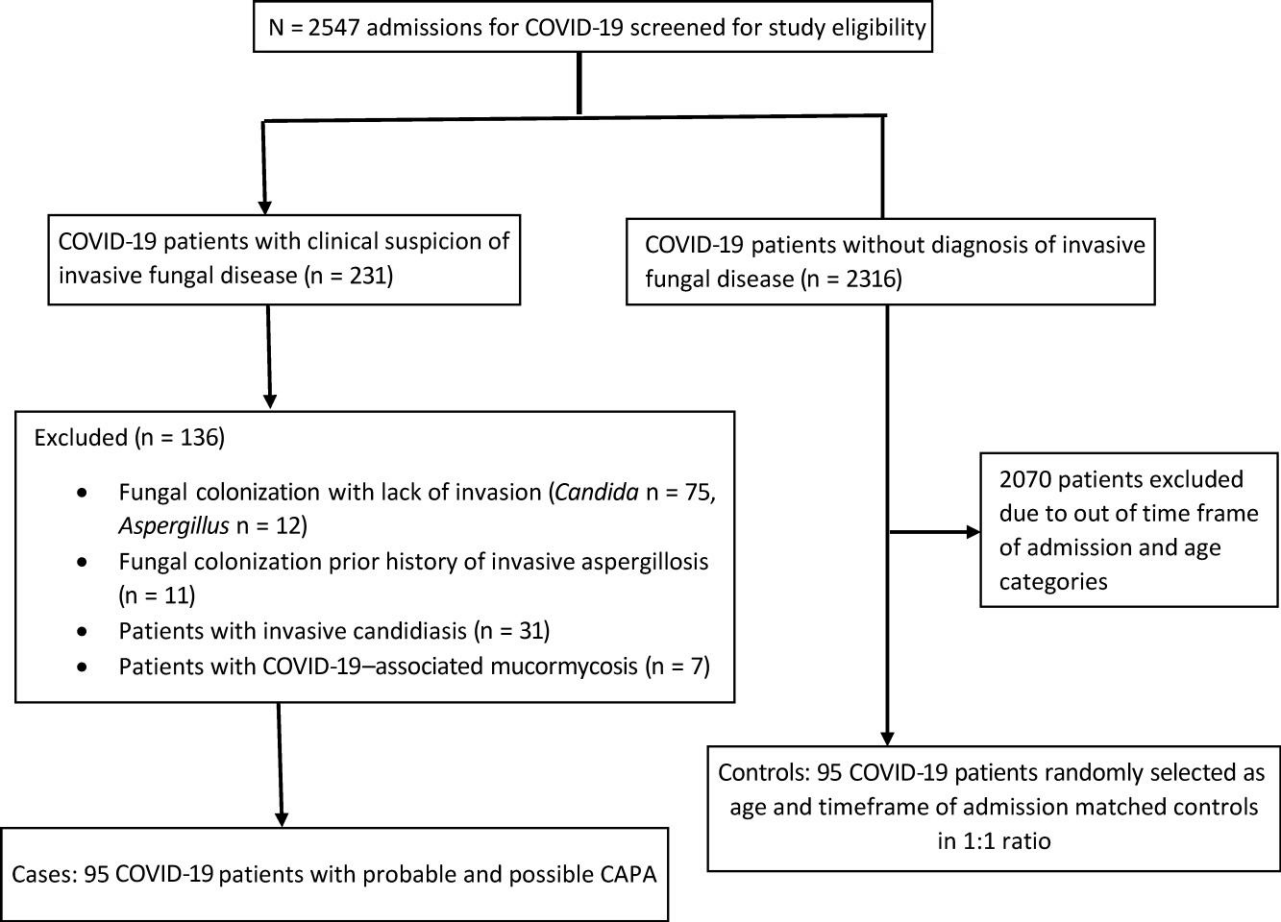
Patient disposition diagram. Abbreviations: CAPA, coronavirus disease 2019–associated pulmonary aspergillosis; COVID-19, coronavirus disease 2019.

We screened 2547 COVID-19 patients for eligibility. Of these, we identified 231 patients with clinical suspicion of invasive fungal infection, and among these, 136 patients were excluded because they had fungal colonization (*Candida/Aspergillus*) in respiratory samples without evidence of invasion, or a prior history of invasive aspergillosis, invasive candidiasis, or COVID-19–associated mucormycosis. However, due to the inherent risk for performing bronchoscopy that existed in our setting during the COVID-19 pandemic, our cohort did not have “proven” cases [14]. The final cohort comprised 95 cases (47 probable and 48 possible) and 95 controls. The patient selection procedure is depicted in the Consolidated Standards of Reporting Trials (CONSORT) diagram shown in Figure 1.

### Baseline Characteristics

Baseline characteristics are depicted in Table 1. At baseline, patients with CAPA had significantly more severe COVID-19 and higher rates of EORTC risk factors, neutropenia, lymphopenia, diabetes mellitus, use of corticosteroids and anticoagulants for COVID-19, use of baricitinib, use of broad-spectrum antibiotics, and use of fluconazole prophylaxis. Of note, there was strong association between lymphopenia and use of immunosuppressants. Among patients with lymphopenia, 89.1% were receiving immunosuppressive drugs, compared to 62.9% of those without lymphopenia (McNemar test, *P* < .01). There was a strong association between use of broad-spectrum antibiotics and CAPA diagnosis, with 93.1% of patients in the case group and 70.5% of patients receiving broad-spectrum antibiotics (McNemar test, *P* < .01). Patients with CAPA also had significantly lower rates of other microbiologically confirmed bacterial infections.

**Table 1.** Baseline Characteristics and Risk Factors of Coronavirus Disease 2019 (COVID-19) Patients With (Cases) or Without (Controls) Possible or Probable COVID-19–Associated Pulmonary Aspergillosis

Variable	No. (%)			
	Total Cases (n = 95)	Controls (n = 95)	Probable (n = 47)	*P* Value^a^
Age, y, mean ± SD	56.03 ± 15.2	54.70 ± 16.3	55.98 ± 14.31	.56
Male sex	74 (78)	66 (69)	34 (72)	.12
Severity of COVID-19				
Mild^b^	11 (12)	37 (39)	5 (11)	<.001
Moderate^c^ to severe^d^	84 (88)	58 (61)	42 (89)	
CAPA disease classification				
Probable	47 (49)	NA	47 (100)	
Possible	48 (51)	NA	…	
Comorbidities				
Diabetic ketoacidosis during hospitalization	1 (1)	4 (4)	1 (2)	.17
Diabetes mellitus present at admission	33 (35)	49 (52)	16 (34)	.028
COPD and asthma	7 (7)	6 (6)	3 (6)	.67
Hypertension	37 (39)	44 (46)	20 (42)	.37
Chronic liver disease	9 (9)	9 (9)	3 (6)	.99
Chronic kidney disease	24 (25)	22 (23)	16 (34)	.86
Risk factors				
Procedure prior to event	2 (2)	3 (3)	0 (0)	.5
Presence of any EORTC risk factors^e^	37 (39)	9 (9)	16 (34)	<.01
Lymphopenia^f^	75 (79)	16 (17)	38 (81)	<.01
Neutropenia^g^	12 (13)	2 (2)	4 (9)	.01
Hematologic malignancy	7 (7)	7 (7)	3 (6)	.99
Transplant^h^	3 (3)	2 (2)	2 (4)	.99
Prolonged steroid use^i^	7 (7)	1 (1)	2 (4)	.06
T-cell and B-cell immunosuppressants	2 (2)	1 (1)	1 (2)	.99
Ibrutinib use	1 (1)	0 (0)	0 (0)	.99
Coronary artery disease	7 (7)	10 (11)	3 (6)	.61
HbA1c, median (IQR), mmol/mol	8.6 (2.9)	7.5 (2.3)	9.7 (2.7)	.11
Lymphocyte count at admission, K/µL, median (IQR)	115.3 (813.0)	267.3 (729.7)	97.4 (216.5)	<.01
Leukocyte count, K/µL, median (IQR)	10.68 (6.49)	11.1 (6.3)	12.3 (6.1)	.918
Culture positive bacterial infection in blood/respiratory secretions	4 (4)	15 (16)	0 (0)	.014
Medications				
Antivirals^j^	78 (82)	68 (72)	39 (83)	.12
Anticoagulants	82 (86)	60 (63)	41 (87)	.001
Corticosteroid therapy for COVID-19	82 (86)	64 (67)	40 (85)	.003
Dexamethasone	65 (68)	59 (62)	37 (79)	.03
Hydrocortisone	6 (6)	2 (2)	3 (6)	.08
Methylprednisolone	4 (4)	2 (2)	0 (0)	.23
Duration of corticosteroid use, d, median (IQR)	7 (12)	7 (8)	6.5 (12)	.039
Vasopressor requirement	36 (38)	17 (18)	16 (34)	.002
Baricitinib	7 (7)	1 (1)	4 (9)	.03
Monoclonal antibody	0 (0)	1 (1)	0 (0)	
Broad-spectrum antibiotics	88 (93)	67 (71)	43 (91)	<.001
Meropenem	44 (46)	22 (23)	23 (49)	<.001
Piperacillin-tazobactam	36 (38)	26 (27)	30 (64)	.06
Ceftriaxone	3 (3)	6 (6)	2 (4)	.51
Azithromycin	25 (26)	19 (20)	25 (53)	.51
Cefoperazone-sulbactam	4 (4)	2 (2)	0 (0)	.2
Other antibiotics^k^	0 (0)	11 (12)	0 (0)	
Antifungal prophylaxis				
Mold-active prophylaxis	5 (5)	3 (3)	0 (0)	.36
Fluconazole prophylaxis	37 (39)	18 (19)	18 (38)	.004

Abbreviations: CAPA, coronavirus disease 2019–associated pulmonary aspergillosis; COPD, chronic obstructive pulmonary disease; COVID-19, coronavirus disease 2019; EORTC, European Organisation for Research and Treatment of Cancer; HbA1c, glycated hemoglobin; IQR, interquartile range; NA, Not Applicable; SD, standard deviation.

^a^ *P* value reflects testing for significant association between total cases and controls only.

^b^No breathlessness or hypoxia.

^c^Dyspnea and/or hypoxia, fever, cough, oxygen saturation ≤94% on room air, respiratory rate ≥24 breaths per minute.

^d^Pneumonia plus 1 of: respiratory rate ≥30 breaths per minute, severe respiratory distress, or oxygen saturation ≤90% on room air.

^e^Individual conditions for EORTC risk factors are not mutually exclusive.

^f^Peripheral blood lymphocyte count <1500 cells/μL.

^g^Absolute neutrophil count <1500 cells/μL.

^h^Includes 1 patient with solid organ transplant and 2 patients with hematopoietic stem cell transplant.

^i^Defined as use of corticosteroids for >3 weeks.

^j^Includes remdesivir and favipiravir.

^k^Includes teicoplanin, vancomycin, colistin.

### Clinical Outcomes

Table 2 shows how time to clinical outcomes is influenced by a positive CAPA diagnosis. The risk for being transferred to ICU significantly increased after a CAPA diagnosis (*P* < .001). Furthermore, a CAPA diagnosis significantly increased the risk for mortality (*P* < .001). On the other hand, a CAPA diagnosis did not significantly increase the risk for requiring mechanical ventilation (*P* = .4).

**Table 2.** Hazard Ratios for the Time Until Mechanical Ventilation, Transfer to Intensive Care Unit, and Death From a Cox Regression Model for the Time-Varying Covariable Coronavirus Disease 2019–Associated Pulmonary Aspergillosis

Outcome	HR (95% CI)	*P* Value
Mechanical ventilation	0.37 (.04–3.07)	.4
ICU	8.47 (4.27–16.8)	<.001
Death	2.96 (1.68–5.20)	<.001

Abbreviations: CI, confidence interval; HR, hazard ratio; ICU, intensive care unit.

### Predictors of CAPA

**Figure 2.**
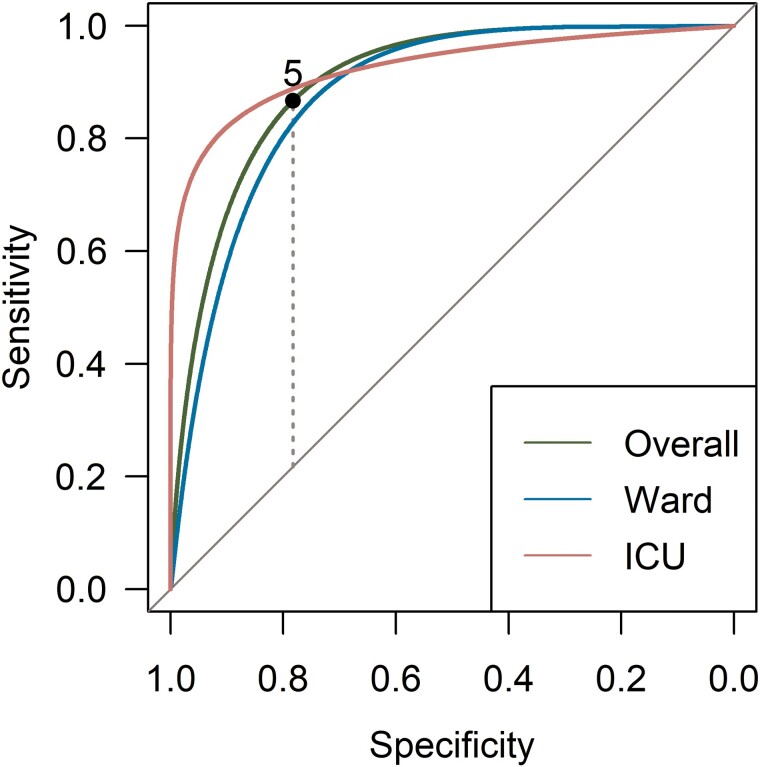
Receiver operating characteristic curve for the coronavirus disease 2019–associated pulmonary aspergillosis risk score. The dotted line indicates the position of the Youden index, with an optimal cut-off point of 5. Abbreviation: ICU, intensive care unit.

Table 3 shows the LASSO model for distinguishing probable cases from their matched controls. All baseline characteristics shown in Table 1 were used as input for the LASSO model. The risk score for a patient is calculated by adding the weighted scores assigned to all risk factors that are present in that patient. A multivariable model identified the following predictors of CAPA (in descending order of weight): lymphopenia, presence of any EORTC risk factor, and broad-spectrum antibiotic use. Isolation of bacterial pathogens in blood or respiratory secretions (BAL/sputum) decreased the likelihood of CAPA. The prediction model had a satisfactory performance in identifying CAPA (Table 4). Figure 2 depicts the receiver operating characteristic of the model and illustrates the selection of an optimal threshold of at least 5 to predict high CAPA risk. This threshold criterion means a high CAPA risk prediction is made under either of 2 conditions: (1) the presence of lymphopenia, EORTC risk factors, and broad- spectrum antibiotic use; or (2) a negative BAL or blood bacterial culture result accompanied by either the sole presence of lymphopenia or the combined presence of EORTC risk factors and broad-spectrum antibiotic use. To further investigate the sensitivity of the model, the model was tested in the subgroup of patients who were initially admitted to ICU. For these patients, the area under the curve of the receiver operating characteristic increased to 85%. To test the dependency of the model on the CAPA classification, the analysis was repeated with possible CAPA added to the control group. For the resulting model, the curve of the receiver operator characteristic decreased to 80%.

**Table 3.** Multivariable Model for the Prediction of Coronavirus Disease 2019–Associated Pulmonary Aspergillosis in Our Cohort

Variable	OR	(95% CI)	*P* Value	Score
Presence of EORTC risk factors	1.99	0.69 (−.60 to 2.23)	.13	3
Lymphopenia	9.41	2.24 (1.92–3.59)	<.01	10
Broad-spectrum antibiotic use	1.79	0.57 (−1.16 to 2.22)	.20	2
Culture-positive bacterial infection in blood/respiratory secretions	0.19	−1.65 (−5.16 to −.30)	.04	−7

Abbreviations: CI, confidence interval; EORTC, European Organisation for Research and Treatment of Cancer; OR, odds ratio.

**Table 4.** Performance Measures of the Coronavirus Disease 2019–Associated Pulmonary Aspergillosis Prediction Model, With 95% Confidence Intervals

Measure	Cross-validation, % (95% CI)
AUROC	82 (79.7–85.3)
Sensitivity	77.4 (76.2–81.2)
Specificity	78.1 (77.1–81.3)
Brier	17.9 (15.8–24.0)
NPV for a cut-off of 5	0.86 (.74–.95)
PPV for a cut-off of 5	0.80 (.74–.95)

Abbreviations: AUROC, area under the receiver operating characteristic curve; CI, confidence interval; NPV, negative predictive value; PPV, positive predictive value.

## DISCUSSION

Our study provides real-world insights for identifying disease characteristics, key predictors of CAPA, and a bedside risk score for CAPA, which is calculated based on the presence of any EORTC risk factor, lymphopenia, broad-spectrum antibiotic use, and absence of bacterial pathogens in microbiological culture.

Recent models to assess the dynamic risk of invasive aspergillosis in different patient populations have included postviral aspergillosis (influenza-associated pulmonary aspergillosis [IAPA], CAPA). These models highlight various risk factors for CAPA that overlap with those for invasive aspergillosis in neutropenia [19]. However, due to the inherent differences between classic neutropenia-related invasive aspergillosis and CAPA, including a reduced propensity for angioinvasion, distinct risk factors for CAPA are likely.

We identified lymphopenia (weighed score of 10) and the use of broad-spectrum antibiotics (weighted score of 2) as risk factors for CAPA, along with the presence of any of the EORTC risk factors (weighted score of 3). Previous studies have identified lymphopenia as a risk factor for invasive mold infections in CAPA [20], IAPA, and other non-CAPA conditions [21]. Lymphopenia as a predictive risk factor for invasive aspergillosis has been attributed to the absence of interferon-γ production by Th1-type lymphocytes, which is an essential pathway of adaptive immune response preventing invasive fungal infection [22]. Additionally, lymphopenia is a marker of compensatory anti-inflammatory response syndrome (CARS), which represents increase in the levels of anti-inflammatory cytokines. Persistence of CARS has been associated with secondary infections in critically ill patients [23]. Although lymphopenia is a surrogate marker for COVID-19 severity [24], our study did not identify an independent association between severity of COVID-19 and CAPA, suggesting that the association of lymphopenia with CAPA is irrespective of COVID-19 severity. Of note, in our study, incidence of CAPA was not limited to patients with severe COVID-19, and a minority of CAPA patients had mild COVID-19.

Many studies have addressed the indiscriminate use of antimicrobials during the COVID-19 pandemic [25, 26], especially in LMICs; these studies emphasized the potential counterintuitive outcomes, such as the emergence of invasive fungal infections. However, direct evidence of increased invasive mold infections resulting from antimicrobial use during the pandemic has not been established. The use of broad-spectrum antibiotics can disrupt normal microbiota, possibly facilitating mold proliferation and, when coupled with other risk factors, favoring invasive fungal disease [27]. Broad-spectrum antimicrobial use was identified as a predictor for CAPA, implicating the need for better antibiotic use strategies. Though our study finds an association between broad-spectrum antimicrobial use in COVID-19 and CAPA, it does not establish causality, emphasizing the need for further prospective validation. Fluconazole use was observed to be higher in cases, primarily administered empirically due to heightened suspicion of invasive fungal infections. The presence of other bacterial pathogens in blood or respiratory samples constituted the only variable with a negative weight in our CAPA prediction model [28]. Coinfections in invasive aspergillosis are largely limited to patients with hematologic malignancies and those who have undergone allogeneic stem cell transplant [29]. However, coinfections and superinfections with bacterial pathogens have been well described in COVID-19, along with the potentiation of pathogenesis and an increased risk of morbidity and mortality [30]. Although CAPA and secondary bacterial infections in a COVID-19 patient may present with similar clinical manifestations, the absence of bacterial pathogens in cultures serves as a potential indicator, necessitating prompt investigation for CAPA. This negative correlation highlights the importance of differentiating between these 2 entities in patients with worsening pneumonia, in whom the lack of detection of bacterial pathogens from either blood or respiratory samples can imply a higher likelihood of CAPA [31].

The lack of association between long-term corticosteroid use and CAPA and the absence of dexamethasone as an independent predictor of CAPA in our scoring system provides an interesting dimension to the existing research, which identifies corticosteroid use as an important risk factor for diagnosis of CAPA. Although the reason for this finding in the current study is unclear, this finding underscores the importance of searching for unique risk factors in each geographical area and the limitations posed by extrapolating data from other studies [32]. Widespread use of corticosteroids, owing to its benefit of reducing mortality rates, in patients belonging to both the case and control groups in the current study could have led to potential bias as well. However, baseline long-term steroid use was uncommon in our cohort. Matching our retrospective cohort for age and period of admission, and conducting further propensity-weighted analysis for COVID-19 severity, revealed higher rates of mortality, adverse outcomes, and ICU shifts in patients with CAPA. It is noteworthy that COVID-19 severity failed to emerge as a risk factor for CAPA in our study, and CAPA complicated the clinical course of even mild COVID-19 infection.

Limitations to this study include that the study was conducted as a monocentric study in a tertiary care academic hospital, and the retrospective design does not allow for causal statements about the identified risk factors. Future prospective trials are required to overcome the challenges posed by the lack of validation of the score and the retrospective study design.

In conclusion, our study offers a simple predictive tool for diagnosis of CAPA in LMICs. By identifying key risk factors and developing a CAPA risk score, we contribute to management strategies for this complex infection.

## Supplementary Material

ofae406_Supplementary_Data

## Supplementary Data

Supplementary materials are available at Open Forum Infectious Diseases online. Consisting of data provided by the authors to benefit the reader, the posted materials are not copyedited and are the sole responsibility of the authors, so questions or comments should be addressed to the corresponding author.

## Notes

***Author contributions.*** D. T. S., M. M., and T. M. J. were involved in conceptualization, manuscript writing, reviewing, and editing. M. A. R., S. G. N., C. V. N., S. S. S., and P. P. were involved in data collection and follow-up. K. G. K., S. S. P., and J. Y. were involved in manuscript writing, literature searching, and references. D. T. S. and M. M. were responsible for project implementation. G. G. and F. E. did the data analysis and were involved in reviewing and editing the manuscript. G. G., F. E., and M. M. prepared all figures in the manuscript. All authors reviewed the manuscript.

***Data availability.*** The datasets used and/or analyzed during the current study are available from the corresponding author on reasonable request.
