# Supplementary material for: Predicting COVID 19–Associated Pulmonary Aspergillosis Risk in Low- and Middle-Income Countries: A Matched Case-Control Study
Source: Open Forum Infect Dis. 2024 Jul 23;11(7):ofae406. doi: 10.1093/ofid/ofae406 (PMC11273325; doi:10.1093/ofid/ofae406)
Supplement: ofae406_Supplementary_Data [file ofae406_supplementary_data.zip › SUPPLEMENTARY MATERIAL 2.docx]

| **Characteristic** | **log(OR)***^1^* | **95% CI***^1^* | **p-value** |
| --- | --- | --- | --- |
| EORTC | 1.8 | -0.28, 4.3 | 0.10 |
| Lymphopenia | 4.8 | 2.6, 8.4 | <0.001 |
| BroadspectrumAntibioticUse | 2.2 | 0.15, 5.2 | 0.065 |
| BloodBALpositivity | 2.6 | 1.1, 4.4 | 0.002 |
| *^1^*OR = Odds Ratio, CI = Confidence Interval |  |  |  |
|  | | | |

Multivariable model for the prediction of COVID-19–associated pulmonary aspergillosis in our ICU patients.
